# Re-engineering a NiFe hydrogenase to increase the H_2_ production bias while maintaining native levels of O_2_ tolerance†
†Electronic supplementary information (ESI) available: Experimental protocol details and Fig. S1–S7. See DOI: 10.1039/c6cc00515b



**DOI:** 10.1039/c6cc00515b

**Published:** 2016-03-31

**Authors:** Lindsey A. Flanagan, John J. Wright, Maxie M. Roessler, James W. Moir, Alison Parkin

**Affiliations:** a Department of Chemistry , University of York , Heslington , York , YO10 5DD , UK . Email: alison.parkin@york.ac.uk ; Tel: +44 1904 322561; b School of Biological and Chemical Sciences , Queen Mary University of London , Mile End Road , London E1 4NS , UK; c Department of Biology , University of York , York YO10 5DD , UK

## Abstract

Naturally occurring oxygen tolerant NiFe membrane bound hydrogenases have a conserved catalytic bias towards hydrogen oxidation which limits their technological value. We present an *Escherichia coli* Hyd-1 amino acid exchange that apparently causes the catalytic rate of H_2_ production to double but does not impact the O_2_ tolerance.

## Abstract 2

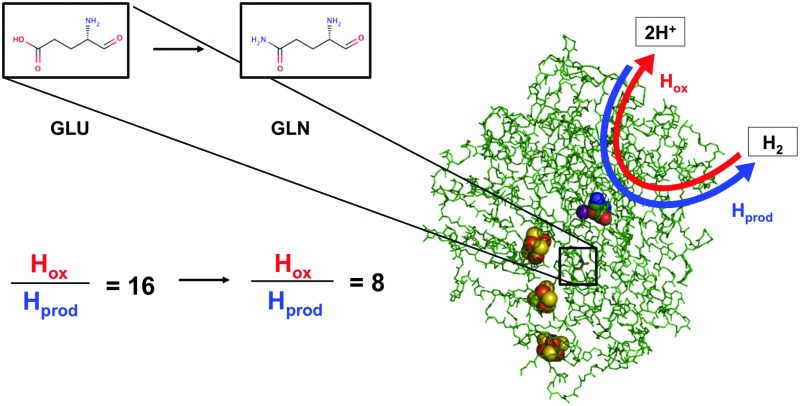
A single site amino acid exchange yields a NiFe hydrogenase with increased bias towards hydrogen production but conserved oxygen tolerance.

Biology offers a source of inspiration for the discovery of highly active, precious-metal-free molecular H_2_ production (proton reduction) catalysts, because many microbes have evolved to produce hydrogen from protons and electrons (2H^+^ + 2e^–^ → H_2_) using enzymes known as hydrogenases.^1,2^ Most nickel-iron hydrogenases, enzymes containing a NiFe bimetallic active site, have limited application as H_2_ catalysts in “one-pot” light-driven water-splitting devices with the O_2_-generating anode and H_2_-producing cathode in same compartment. This is because O_2_ binds at the active site to produce the catalytically inactive Ni–A species (such inhibition is described as “O_2_ sensitivity”). There is a well-studied subclass of NiFe hydrogenases which retain catalytic activity in O_2_, these are the membrane-bound, O_2_ tolerant NiFe hydrogenases (O_2_-tolerant MBH).^3^ Unfortunately, the evolution of O_2_ tolerance was concomitant with the evolution of a bias towards H_2_ oxidation.^3^ In this study we have rationally redesigned an O_2_-tolerant MBH to yield a catalyst with an increased H_2_ production: H_2_ oxidation bias but native levels of O_2_ tolerance.

NiFe MBHs contain a NiFe active site which is buried within a “large” protein subunit and three iron sulfur (FeS) clusters ligated by a “small” protein subunit.^1,2^ The FeS clusters act as an electron transfer conduit, mediating the flow of electrons between the protein surface of the small subunit and the buried active site. While the active site binding pocket is remarkably well conserved in all NiFe hydrogenases, it is plasticity in the FeS relay which appears to control both the catalytic bias (the ratio of H_2_ oxidation to production activity) and O_2_ sensitivity in NiFe MBH.^2^

All O_2_ tolerant MBH contain an unusual Fe_4_S_3_ “proximal” (closest to the active site) cluster, while the O_2_ sensitive MBH have a standard Fe_4_S_4_ centre in the same position.^2,4–6^ The “medial” (middle of the relay) FeS centre is always a Fe_3_S_4_ centre but despite the structural invariance this centre still plays a vital role in enabling O_2_ tolerant MBH to catalyse H_2_ oxidation in the presence of O_2_.^7^ Armstrong and co-workers have hypothesised that the “distal” (furthest from the active site) FeS cluster controls the thermodynamic driving force which is needed to induce catalysis in a MBH.^8–10^ One of the reasons that the proximal cluster was disregarded as an important tuning point for catalysis is because previous mutations within both *Ralstonia* MBH and *Escherichia coli* hydrogenase-1 were not noted to increase the H_2_ production of these O_2_ tolerant hydrogenases.^5,11^ However, other researchers have shown that the rate of catalysis in a [NiFe] hydrogenase can be altered by changes to the proximal cluster. An increase in the cellular level of H_2_ production by the cyanobacteria *Nostoc punctiforme* accompanies the replacement of the Fe_4_S_4_ proximal cluster of the O_2_-sensitive HupSL uptake hydrogenase with a Fe_3_S_4_ cluster.^12^ Conversely, a drop in the H_2_ production activity of *Desulfovibrio fructosovorans* variants^13^ has been attributed to single site amino acid changes having an impact on the proximal cluster.^8^

*Escherichia coli* (*E. coli*) produce two different membrane-bound hydrogenases, the O_2_ tolerant hydrogenase-1 (Hyd-1) and the O_2_ sensitive hydrogenase-2 (Hyd-2).^14^ Sequence comparisons between *E. coli* Hyd-1 and Hyd-2 can help suggest which amino acid residues play a vital role in controlling how a hydrogenase reacts with substrate and/or inhibitors. The conserved presence of a glutamine (Q) in a position between the active site and proximal cluster of all O_2_ sensitive hydrogenases (position 73 using *E. coli* Hyd-1 numbering), contrasts with the occurrence of a glutamic acid (E) in the same position in most O_2_ tolerant enzymes (Fig. 1). In previous work^15^ on *Salmonella* Hyd-5 we found that an E73A variant sustained lower levels of H_2_ oxidation activity in the presence of O_2_ when compared to Native enzyme, although the O_2_ inhibition remained substantially reversible. Since the mechanism of O_2_ tolerance relies on electron transfer between the proximal cluster and the active site,^4–6^ we hypothesised that E73 might influence the proximal cluster redox potential. As a result of this, we have explored how an E73Q amino acid exchange in *E. coli* Hyd-1 impacts the enzyme's reactivity with both H^+^ and H_2_ substrates and the inhibitor O_2_.

**Fig. 1:**
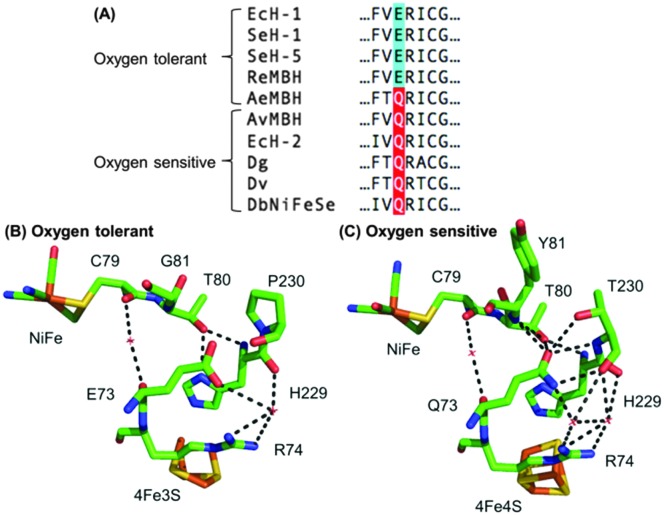
(A) Sequence alignment showing the conservation of large subunit residue 73 amongst oxygen tolerant and oxygen sensitive NiFe hydrogenases. Ec = *E. coli*; Se = *Salmonella enterica*; Re = *Ralstonia eutropha*; Ae = *Aquifex aeolicus*; Av = *Allochromatium vinosum*; Dg = *Desulfovibrio gigas*; Dv = *Desulfovibrio vulgaris*; Db = *Desulfomicrobium baculatum*. (B) Crystal structures of (i) an oxygen tolerant MBH (PDB: ; 3RGW) and (ii) an oxygen sensitive NiFe hydrogenase (PDB: ; 4UD2), showing the location of residue 73 relative to the active site and the proximal cluster. The H-bonding capacity of residue 73 is denoted by the dashed black lines. *E. coli* numbering is used throughout.

Methylene blue (MB) assays in H_2_-saturated buffer were used to compare the enzyme turnover rate of purified E73Q and Native enzyme at pH 4.5, 25 °C.^16^ Very similar H_2_ oxidation rates were measured for both hydrogenases (Native: 21 ± 4 s^–1^; E73Q: 22 ± 3 s^–1^), suggesting that the amino acid exchange does not impact on the enzyme's ability to catalyse H_2_-uptake at the MB redox potential (voltammetry measurements (data not shown) determined *E*_mid_(MB) = +0.113 V *vs.* SHE at pH 4.5, 25 °C). Protein film electrochemistry experiments (Fig. 2A and B) were then performed to explore the enzyme activity over a wider potential range and under different levels of H_2_. To further facilitate the comparison of H_2_ production activity, Fig. 2C shows overlay plots of the data in Fig. 2A and B in the region –0.45 to –0.15 V *vs.* SHE after background electrode charging currents have been removed by averaging the current measured in forward and back scan sweeps.

**Fig. 2:**
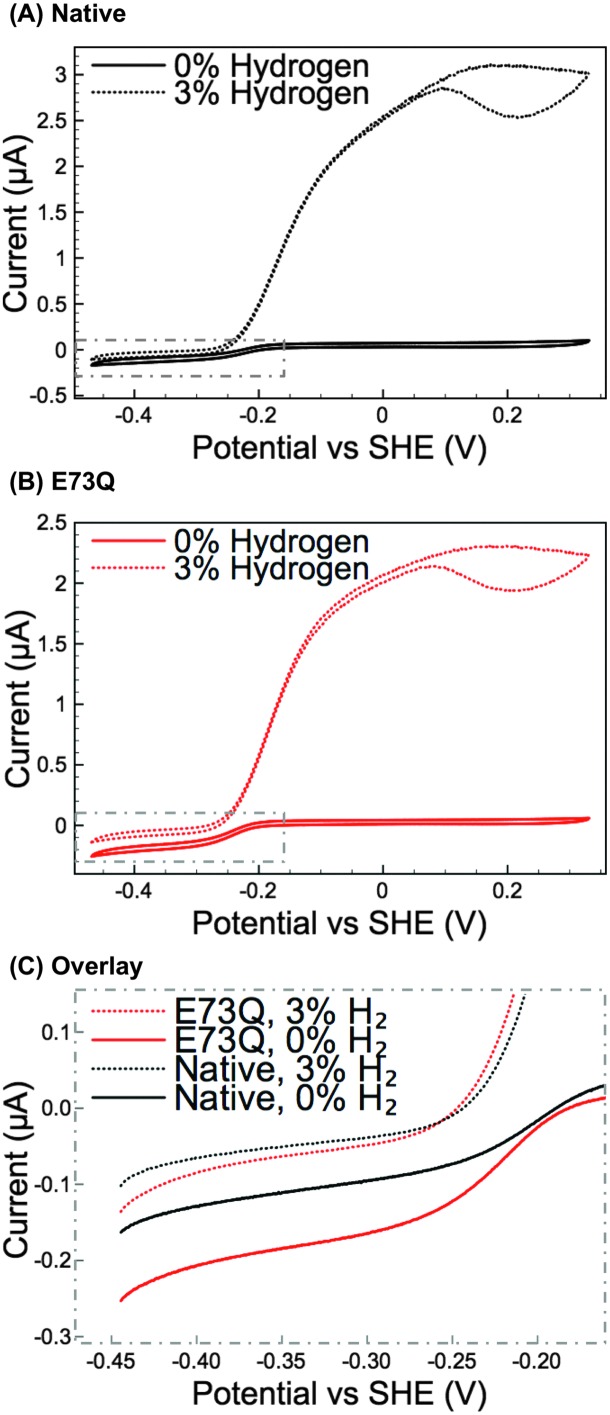
Cyclic voltammetry (CV) measurements at 5 mV s^–1^ of (A) Native *Escherichia coli* hydrogenase-1, and (B) the E73Q variant under gas atmospheres of both 3% H_2_ in N_2_ (dotted lines) and 0% H_2_ (100% N_2_, solid lines). Other experimental conditions: pH 4.5; 37 °C; electrode rotation rate 3500 rpm; carrier gas N_2_ and total gas flow rate 100 scc min^–1^. (C) The difference in H_2_ production activity is emphasised through an overlay of the averaged forward and back scan sweeps of the CVs in the top two panels, the selected region is highlighted by the dotted grey boxes in A and B.

From Fig. 2A and B, comparing the ratio of the maximum positive (H_2_ oxidation) current measured under 3% H_2_ to the maximum negative (H^+^ reduction/H_2_ production) current measured under 0% H_2_ immediately reveals that the catalytic bias of the E73Q variant is significantly different to that of Native enzyme. A value of approx. 16 is generated from calculating *i*_max_(H_2_ oxidation, 3% H_2_) ÷ *i*_max_(H_2_ reduction, 0% H_2_) for Native enzyme, whereas the equivalent ratio for E73Q is approx. 8. If we assume that the rate of H_2_ oxidation is unaffected by the amino acid exchange, then this means that the rate of catalytic H_2_ production by E73Q is approximately double that of Native enzyme.

Although the E to Q amino acid exchange has a dramatic impact on the catalytic activity, the oxygen tolerance remains unchanged. Fig. 3 shows that for both Native and E73Q hydrogenase the same proportion of H_2_ oxidation current is sustained at +0.113 V *vs.* SHE when the experimental headgas is changed from 3% H_2_ in N_2_ to 3% H_2_, 3% O_2_ and 94% N_2_. When the inhibitory O_2_ is removed from the gas flow, both enzymes rapidly recover almost the full extent of their pre-O_2_ activity, demonstrating characteristic “O_2_ tolerant” behaviour. We therefore suggest that we have artificially created an *E. coli* Hyd-1 variant with twice the rate of H_2_ production of the Native enzyme, but equal levels of O_2_ tolerance.

**Fig. 3:**
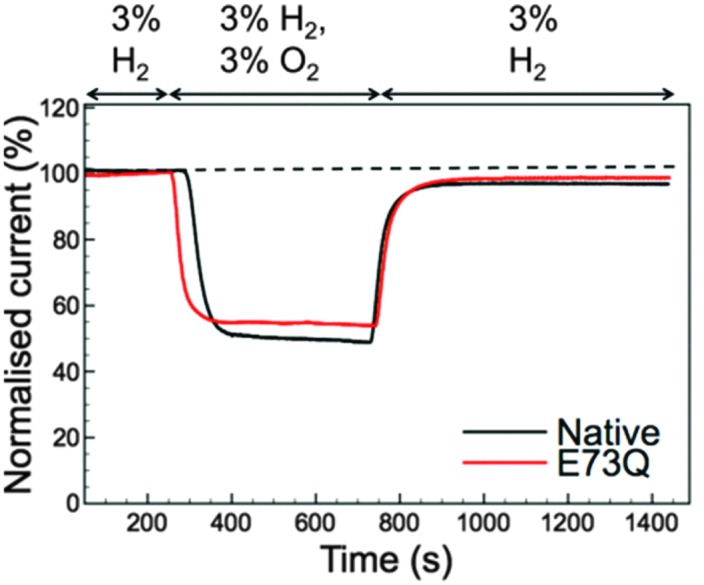
Inhibition and recovery from 3% O_2_ measured at +0.113 V *vs.* SHE with 3% H_2_ throughout. The black line shows data for Native *Escherichia coli* Hyd-1 while the red line depicts the measurement for the E73Q variant. Other experimental conditions: pH 4.5; 37 °C; electrode rotation rate 3500 rpm; carrier gas N_2_ and total gas flow rate 100 scc min^–1^.

In order to probe the origin of the novel reactivity of E73Q, molecular biology was used to create *E. coli* strains to encode the Hyd-1 variants E73K, E73N and E73A (see Fig. S1, ESI†). Unfortunately, only very low levels of the proteins E73K and E73N could be purified (see Fig. S2, ESI†). Electrochemical analysis of E73A showed the same phenotype as was previously observed for the *Salmonella* Hyd-5 variant,^15^ namely Native-like catalytic activity and increased sensitivity to O_2_ (see Fig. S3, ESI†). Dance^17^ has rationalised that the loss of O_2_ tolerance in a *Salmonella* Hyd-5 E73A variant is attributable to the decoupling of the proton transfer network between the active site and proximal cluster. This decoupling was proposed to arise because the non-polar alanine is incapable of proton donation/acceptance. However, our E73Q variant is O_2_ tolerant despite the fact that glutamine does not have an acidic or basic side chain. Instead, a glutamine in this position would be expected to participate in extensive H-bonding, based on the structure of O_2_ sensitive hydrogenases (Fig. 1). We therefore hypothesise that the critical function of residue 73 in the O_2_ tolerance mechanism is to participate in stabilising the H-bonding interaction between the active site and the proximal cluster (Fig. 1), and a direct role in proton exchange is not required.

Modifications to a NiFe hydrogenase gas channel have previously been demonstrated to impact on the reactivity of the enzyme,^1^ but there is no evidence that the catalytic bias of E73Q can be ascribed to a gas channel effect. Firstly, Fig. 3 suggests that O_2_ access to the NiFe active site is unaffected by the E to Q amino acid exchange. Further experiments also suggest that H_2_ movement through the enzyme is not altered in E73Q relative to Native enzyme. Using data extracted from cyclic voltammograms measured under different percentages of H_2_ (Fig. S4, ESI†) permits a Hanes Woolf analysis to be conducted (Fig. S5A, ESI†). The Michaelis constant for H_2_, *K*_M_(H_2_), at +0.113 V *vs.* SHE is thus calculated for Native and E73Q, yielding very similar values of 4.2 ± 1.0 μM and 3.8 ± 1.7 μM, respectively (average value ± standard error, *n* = 4). The product inhibition constant (*K*_i_) for H_2_ production at –0.285 V *vs.* SHE has also been analysed (Fig. S5B, ESI†), for Native enzyme and E73Q, with 9.5 ± 1.7 μM for the former and 4.6 ± 0.8 μM for the latter. This difference is to be expected, since the variants have similar H_2_ production activity under 3% H_2_ despite the greater H_2_ production activity of E73Q under 0% H_2_ (Fig. 2C).

Since the H-bonding network around E73 in an O_2_ tolerant NiFe MBH is different to that around the equivalent Q in an O_2_ sensitive enzyme (Fig. 1), we also explored whether changing the pH revealed any characteristic differences between Native and E73Q hydrogenase. As shown in Fig. S6, ESI,† apart from the enhanced H_2_ production activity of E73Q enzyme, no substantial change in the pH profile is detected, including in the high potential region where reversible formation of the “Ni–B” OH^–^ bound Ni(iii) oxidised inactive state, is observed. In order to further confirm this, the experiments shown in Fig. S7, ESI,† were conducted, permitting extraction of the potential of maximum rate of reactivation, Eswitch, which is the same for both enzymes, as shown in Fig. S8, ESI.† It is therefore difficult to rationalise the changes in catalytic bias in light of proton availability.

Following the careful elimination of substantial gas or proton transfer pathway changes as the possible origin of changes in reactivity, it is logical to speculate that changes to the iron–sulfur relay underlie the differences between the catalytic bias of the E73Q and Native hydrogenases. We have explored this by carrying out an electron paramagnetic resonance (EPR) redox titration^18^ on E73Q (Fig. 4 and Fig. S9, ESI†), monitoring the signal corresponding to EPR-visible “super-oxidised” state of the proximal cluster. This has yielded a midpoint potential of +211 ± 10 mV at pH 7 for the transition of the proximal cluster between the super-oxidised state at very high potentials and the EPR-silent “oxidised” state at intermediate potentials ([Fe_4_S_3_Cys_2_]^3+^ → [Fe_4_S_3_Cys_2_]^2+^ + 1e^–^). The potential for this proximal-cluster process is very similar to that previously reported for Native hydrogenase, +230 ± 15 mV at pH 6.^19^ We consider that it is appropriate to compare these midpoint potential values despite the difference in pH because it has been shown for the *Aquifex aeolicus* MBH that the high-potential redox transition of the proximal cluster is not affected by pH over the range 6.4 to 7.4, and pH-related changes to the lower potential [Fe_4_S_3_Cys_2_]^2+/1+^ redox couple only occur at pH > 7.^20^

**Fig. 4:**
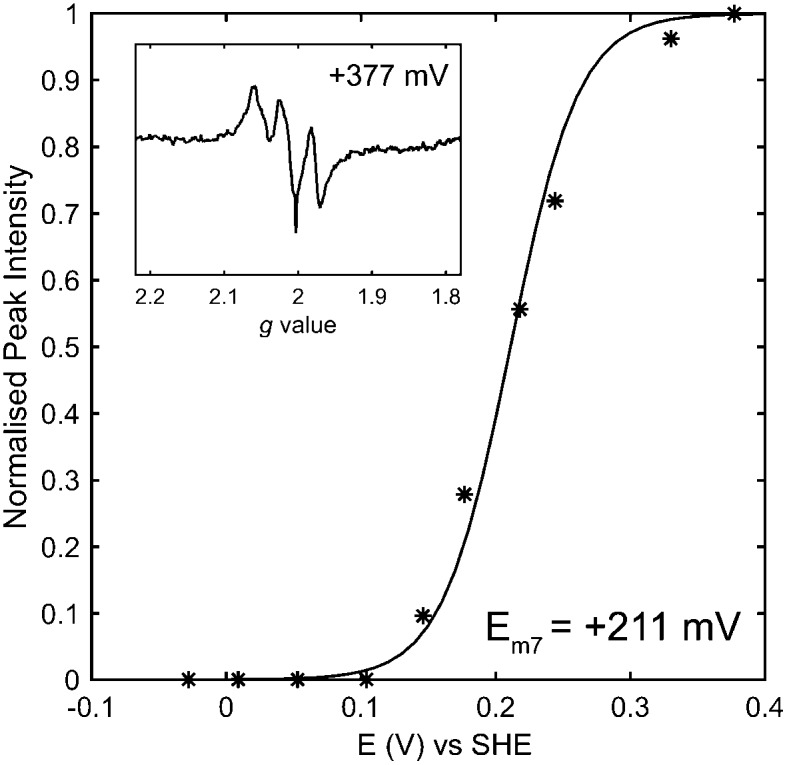
Potentiometric titration of Hyd-1 E73Q. The *g* = 1.97 peak of the [Fe_4_S_3_Cys_2_]^3+^ EPR signal (inset) was monitored as a function of potential and fitted to the one-electron Nernst equation (solid line, *R* = 0.9923).

The development of the medial cluster EPR signal as a function of potential (Fig. S9, ESI†) also correlates with previous measurements of Native *E. coli* Hyd-1, suggesting that the midpoint potential of this cluster is unaffected by the E73Q amino acid exchange. (Unfortunately a quantitative analysis of the redox potential of spectroscopic transitions at lower potential than the proximal cluster was not possible due to the low protein concentration). Given the large distance between E73 and the distal cluster,^21^ we predict that this FeS centre (invisible in EPR)^19^ will also have a redox potential which is the same in both the E73Q variant and Native enzyme. The changes in catalytic bias of E73Q therefore cannot be interpreted in light of changes to the energetics of the electron transfer relay.

Based on the evidence above, we speculate that the E73Q amino acid exchange impacts on the active site chemistry of Hyd-1. Fourier transform InfraRed spectroelectrochemical experiments, beyond the scope of this study, would be the logical suggestion for testing this hypothesis.

In conclusion, we have confirmed that it is possible to significantly perturb the *in vitro* catalytic bias of an O_2_ tolerant NiFe MBH while maintaining the native levels of resistance to aerobic inhibition. We have engineered an apparent doubling of H_2_ production rates through altering an amino acid residue which is located close to the proximal cluster. We have followed a reverse engineering strategy suggested by comparing *E. coli* Hyd-1 and Hyd-2, emphasising the utility of working with bacteria which express multiple hydrogenases.

The research leading to these results has received funding from the Wellcome Trust through the Combating Infectious Disease: Computational Approaches in Translational Science (CIDCATS WT095024MA) CDT program, from a Royal Society Research Grant Award (RG2014R2) to AP and from EP/M024393/1 (to MMR) and EP/M506394/1 (supporting JJW).
