# Supplementary material for: Re-engineering a NiFe hydrogenase to increase the H2 production bias while maintaining native levels of O2 tolerance
Source: Chem Commun (Camb). 2016 Mar 31;52(58):9133–6. doi: 10.1039/c6cc00515b (PMC5040170; doi:10.1039/c6cc00515b)
Supplement: Supplementary file 1 [file CC-052-C6CC00515B-s001.pdf]

ESI for Manuscript "**Re-engineering a NiFe hydrogenase to increase the H_2 production bias while maintaining native levels of O_2 tolerance**"

Lindsey A. Flanagan,[a] John J. Wright,[b] Maxie M. Roessler,[b] James W. B. Moir[c] and Alison Parkin[a,*]

[a.] Department of Chemistry, University of York, Heslington, York, YO10 5DD, UK, E-mail: alison.parkin@york.ac.uk; Tel: +44 1904 322561

[b.] School of Biological and Chemical Sciences, Queen Mary University of London, Mile End Road, London E1 4NS, UK.

[c.] Department of Biology, University of York, York YO10 5DD, UK.

1. Molecular biology procedures to generate *Escherichia coli* hydrogenase-1 variants

Escherichia coli hydrogenase variants were created by making chromosomal mutations rather than via overexpression from plasmids. This is the standard approach, as used by other groups.[1] This approach has been taken because NiFe hydrogenase assembly is a multicomponent process which is not yet fully understood,[2-4] and it is therefore difficult to construct plasmids which contain the full complement of necessary genes.

In order to make the *Escherichia coli* strains used in this study, chromosomal mutations were created using a modified version of the streptomycin counter-selection protocol detailed by Heermann et al.[5] This is the first time such a method has been used for manipulation of hydrogenases. In the original protocol,[5] electroporation is used to transform DNA into *E. coli*, but we used chemically competent *E. coli* cells.[6] *E. coli* strains were cultured in LB media and on LB agar plates containing ampicillin (50 µg/ml) to select for the presence of the pRed/ET plasmid, kanamycin (50 µg/ml) to select for the presence of the *rpsL-neo* cassette and streptomycin (50 µg/ml) to select for the absence of the *rpsL-neo* cassette.

More, precisely, our stepwise protocol proceeded as follows:

i. *E. coli* W3110 was made streptomycin resistant (LAF-001) by insertion of the *rpsL*150 allele from *E. coli* MC1061. The *rpsL*150 from *E. coli* MC1061 was amplified by PCR (with GoTaq polymerase, Promega) using primers rpsl_up1 and rpsl_down1 (Table S1), and transformed into *E. coli* W3110 containing the pRed/ET^{amp} plasmid (obtained from the "Quick and Easy *E. coli* Gene Deletion Kit" (Cambio)).

ii. Strain LAF-007 (E73Q) was generated directly from LAF-001 via a four-part process:
   a. The *rpsL-neo* cassette (template DNA was obtained from the "Counter-Selection BAC Modification Kit" (Cambio)) flanked by *hyaB* sequences (amplified using the rpsL-neo primers hyaBe73q_neo_sense and hyaBe73q_neo_antisense and Q5

polymerase, NEB), was inserted into the *hyaB* gene, giving strain LAF-004.

b. The *rpsL-neo* cassette was replaced by a version of *hyaB* containing DNA encoding for the E73Q mutation (amplified with the “overlap” primers hyaBe73q_olap_sense and hyaBe73q_olap_antisense and Q5 polymerase, NEB). This gave strain LAF-005.
c. The *rpsL-neo* cassette (template DNA was obtained from the "Counter-Selection BAC Modification Kit" (Cambio)) flanked by *hyaA* sequences (amplified using the rpsL-neo primers hyaA_histag_neo_sense and hyaA_his2_neo_antisense and Q5 polymerase, NEB), was inserted into the *hyaA* gene to give strain LAF-006.
d. The *rpsL-neo* cassette was replaced by a version of *hyaA* containing DNA encoding a C-terminal polyhistidine tag (amplified with the “overlap”primers hyaA_his2_olap_sense and hyaA_his2_olap_antisense and Q5 polymerase, NEB), yielding strain LAF-007.

iii. A strain used to express “Native” enzyme, containing polyhistidine tagged *E. coli* hydrogenase-1 (strain LAF-003), was constructed from strain LAF-001 using the latter two processes from step ii.
iv. The LAF-003 strain was used as the parent strain to generate LAF-008 strain (with the *rpsL-neo* cassette inserted into *hyaB* close to the coding region of amino acid 73 using primers hyaBe73a_neo_sense and hyaBe73a_neo_antisense). Strain LAF-008 was then mutated to give LAF-009 (E73A), LAF-010 (E73K) and LAF-011 (E73N) via removal of the cassette using the relevant overlap primers (Table S1).

Successful insertion of the correct single site mutation was verified by amplification of the region of the chromosome and sequencing (GATC Biotech) as shown in Fig. S1. Table S1 lists full details of all the primers, and Table S2 gives the strain information.

LAF-003 (E)
LAF-007 (Q)
LAF-009 (A)
LAF-010 (K)
LAF-011 (N)

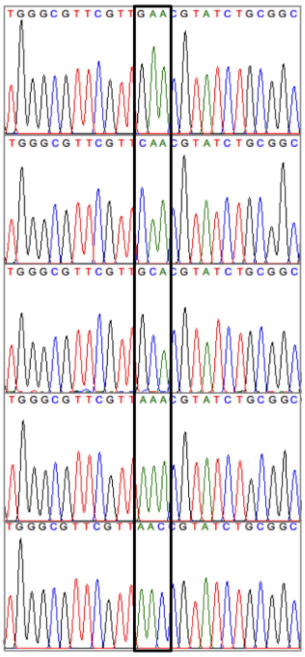

Fig. S1. Sequence of the chromosomes for each strain confirming insertion of the correct single site mutations, as visualized with the FinchTV program (Geospiza). Black rectangle illustrates the codon corresponding to position 73 of the Hyd-1 Large subunit.

Table S1 Primers used in this study. All oligonucleotides, were obtained from Sigma Aldrich in a salt-free grade.

Stage	Function	Name	Sequence (5'-3')
Amplification of *rpsL* gene		rpsl_up1	CTTGACACCTTTTCGGCATCGC
		rpsl_down1	CGTTGTTAATTCAGGATTGTCC
Insertion of polyhistidine tag	rpsL-neo primers	hyaA_histag_neo_sense	CCAGCAACCTACAGAAACCGAACATCAGCCAGGCAATGAGGATAAACAGGCACATCATCATCATCATCATTGAGGCCTGGTGATGATGGC GGGATCG
		hyaA_his2_neo_antisense	GGCGGCGTCCGGCATTATTGATGGTGTATCCCTGAGTTTCGTACTGAGTGCTCATGCCTGTTTATCCTCATTTCAGAAGAACTCGTCAAGAAGG CG
	overlap primers	hyaA_his2_olap_sense	TACAGAAACCGAACATCAGCCAGGCAATGAGGATAAACAGGCACATCATCATCATCATCATTGAAATGAGGATAAA
		hyaA_his2_olap_antisense	TCCGGCATTATTGATGGTGTATCCCTGAGTTTCGTACTGA GTGCTCATGCCTGTTTATCCTCATTTCAATGATGATGATGATG
Creation of *hyaB* E73Q mutation	rpsL-neo primers	hyaBe73q_neo_sense	GCTGGAGATCATCCTACAAGGGCGCGACCCGCGCGATGCGTGGGCGTTCGTTGGCCTGGTGATGATGGCGGGATCG
		hyaBe73q_neo_antisense	GATGGCGTAAACCGAAGCCAGGGCGTGTACGCCAGTACAGACGCCGCAGATACGTTGTCAGAAGAACTCGTCAAGAAGGCG
	overlap primers	hyaBe73q_olap_sense	GCTGGAGATCATCCTACAAGGGCGCGACCCGCGCGATGCGTGGGCGTTCGTTCAACGTATCTG
		hyaBe73q_olap_antisense	GCGTAAACCGAAGCCAGGGCGTGTACGCCAGTACAGACGCCGCAGATACGTTGAACGAACGCCCAC
Creation of *hyaB* E73A mutation	rpsL-neo primers	hyaBe73a_neo_sense	GCTGGAGATCATCCTACAAGGGCGCGACCCGCGCGATGCGTGGGCGTTCGTTGGGCCTGGTGATGATGGCGGGATCG
		hyaBe73a_neo_antisense	GATGGCGTAAACCGAAGCCAGGGCGTGTACGCCAGTACAGACGCCGCAGATACGTGTCAGAAGAACTCGTCAAGAAGGCG
	overlap primers	hyaBe73a_olap_sense	GCTGGAGATCATCCTACAAGGGCGCGACCCGCGCGATGCGTGGGCGTTCGTTGCACGTATCTG
		hyaBe73a_olap_antisense	GCGTAAACCGAAGCCAGGGCGTGTACGCCAGTACAGACGCCGCAGATACGTGCAACGAACGCCCAC
Creation of *hyaB* E73K mutation	overlap primers	hyaBe73k_olap_sense	GCTGGAGATCATCCTACAAGGGCGCGACCCGCGCGATGCGTGGGCGTTCGTTAAACGTATCTG
		hyaBe73k_olap_antisense	GCGTAAACCGAAGCCAGGGCGTGTACGCCAGTACAGACGCCGCAGATACGTTTAACGAACGCCCAC
Creation of *hyaB* E73N mutation	overlap primers	hyaBe73n_olap_sense	GCTGGAGATCATCCTACAAGGGCGCGACCCGCGCGATGCGTGGGCGTTCGTTAACCGTATCTG
		hyaBe73n_olap_antisense	GCGTAAACCGAAGCCAGGGCGTGTACGCCAGTACAGACGCCGCAGATACGGTTAACGAACGCCCAC
Checking and sequencing		counter_hyaA_hisdTM_sense	TCGTTCTACAGCCGCGTGGTC
		forseq_hyaB_H229A_antisense	AGGCGTTCCATATTGACTGCC

Table S2 *Escherichia coli* strains generated in this study.

Strain name	Genotype	Reference
W3110	F¯ lambda¯ *IN(rrnD-rrnE)1 rph-1* (StrepS)	7
MC1061	araD139 Del(araA-leu)7697 Del(lac)X74 galK16 galE15(GalS) lambda¯ e14¯ mcrA0 relA1 rpsL150 spoT1 mcrB1 hsdR2 (StrepR)	8
LAF-001	F¯ lambda¯ *IN(rrnD-rrnE)1 rph-1 rpsL150* (StrepR)	This work
LAF-002	F¯ lambda¯ *IN(rrnD-rrnE)1 rph-1 rpsL150 hyaA(histag):rpsL-neo* (KanR StrepS)	This work
LAF-003	F¯ lambda¯ *IN(rrnD-rrnE)1 rph-1 rpsL150 hyaA(histag)* (StrepR)	This work
LAF-004	F¯ lambda¯ *IN(rrnD-rrnE)1 rph-1 rpsL150 hyaB(E73Q):rpsL-neo* (KanR StrepS)	This work
LAF-005	F¯ lambda¯ *IN(rrnD-rrnE)1 rph-1 rpsL150 hyaBE73Q* (StrepR)	This work
LAF-006	F¯ lambda¯ *IN(rrnD-rrnE)1 rph-1 rpsL150 hyaBE73Q hyaA(histag):rpsL-neo* (KanR StrepS)	This work
LAF-007	F¯ lambda¯ *IN(rrnD-rrnE)1 rph-1 rpsL150 hyaBE73Q hyaA(histag)* (StrepR)	This work
LAF-008	F¯ lambda¯ *IN(rrnD-rrnE)1 rph-1 rpsL150 hyaA(histag) hyaB(E73A):rpsL-neo* (KanR StrepS)	This work
LAF-009	F¯ lambda¯ *IN(rrnD-rrnE)1 rph-1 rpsL150 hyaA(histag) hyaBE73A* (StrepR)	This work
LAF-010	F¯ lambda¯ *IN(rrnD-rrnE)1 rph-1 rpsL150 hyaA(histag) hyaBE73K* (StrepR)	This work
LAF-011	F¯ lambda¯ *IN(rrnD-rrnE)1 rph-1 rpsL150 hyaA(histag) hyaBE73N* (StrepR)	This work

2. Protein purification protocol

The purification protocol is modified from Lukey *et al*.[9] 10 mL starter cultures were grown overnight in LB plus streptomycin (50 µg mL^{-1}) at 37 °C with shaking. Of this, 3 mL was used to inoculate 6 L prewarmed bottles of LB, each containing 1 % glycerol, 0.4 % sodium fumarate, and 50 µg mL^{-1} streptomycin. These were grown overnight at 37 °C until an OD of ~1 was reached.

The cells were harvested by spinning at 5500 *g* for 15 min on a Beckman Avanti centrifuge (JA10 rotor) at 4 °C. Pellets (~50 g total) were resuspended in 125 mL chilled buffer (100 mM Tris pH 7, 300 mM NaCl) before the addition of sucrose (20% w/v) followed by stirring for >45 min at 4 °C. This mixture was then centrifuged at 6500 *g* for 20 min on a Beckman Avanti centrifuge (JA25.50 rotor) at 4 °C. The supernatant was once again discarded and the pellet was resuspended in 300 mL ice-cold pure water (Purite). This mixture was stirred in the fridge for 30 min before making the solution up to 100 mM Tris pH 7, 300 mM NaCl and 9% Triton X-100 and adding EDTA-free protease inhibitors, DNAse and lysozyme. This solution was stirred overnight in the fridge to facilitate solubilisation. The mixture was then divided between 150 mL sample pots and each aliquot was sonicated on ice for 10 x 30 s. The mixture was once again combined and centrifuged at 20000 *g* for 30 min on a Beckman Avanti centrifuge (JA25.50 rotor) at 4 °C. The supernatant was collected and diluted to 600 mL with 100 mM Tris pH 7, and the mixture was made up to 50 mM imidazole in preparation for loading onto the column.

The solubilised protein was loaded onto a 5 mL HisTrap Ni affinity column (GE Healthcare) using an Akta Start, the column was washed with 8 column volumes of buffer A (100 mM Tris, 150 mM NaCl, 50 mM imidazole, pH 7) and protein was eluted using a gradient elution (0-100% buffer B: 100 mM Tris, 150 mM NaCl, 1 M imidazole, pH 7). Presence of hydrogenase enzyme was confirmed using 10% SDS-PAGE (see Fig. S2). Fractions were dialysed overnight in the fridge (100 mM Tris pH7, 150 mM NaCl) in 0.2 micron dialysis tubing. An Amicon Ultra centrifugal filter with 30 kDa cutoff (Merk Millipore) was used to concentrate the protein.

A full spectrum of the purified enzymes (250-700 nm) was obtained on a UV-1061 (Shimadzu) UV-Vis spectrophotometer using a Semi-Micro Cell 108B-QS cuvette (Hellma Analytics). The concentration of the protein was calculated from the A_{280} using the molar extinction coefficient 171,335 $M^{-1}cm^{-1}$ as determined using the ExPASy ProtParam tool.[10]

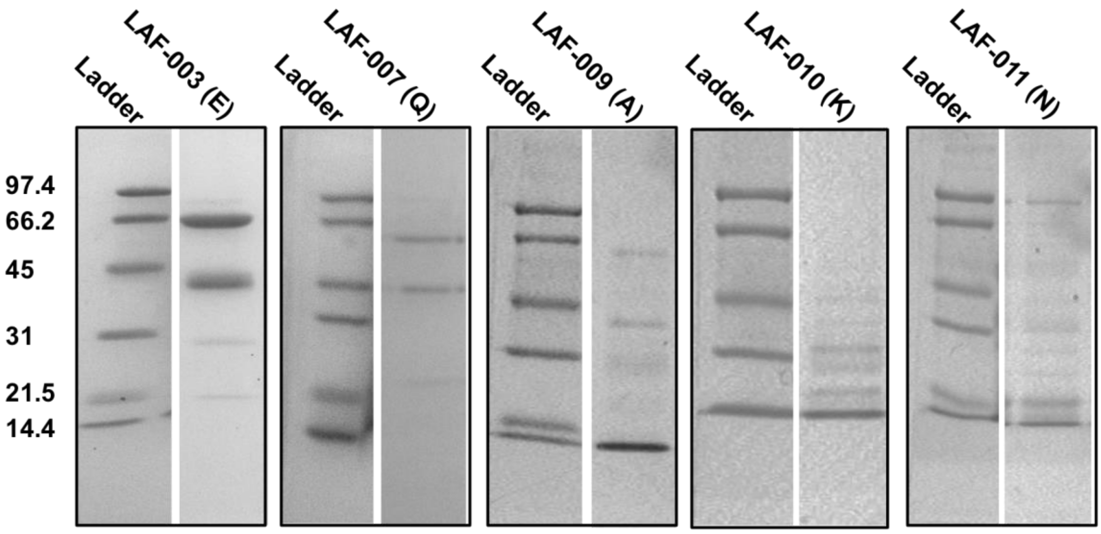

Fig. S2. SDS-PAGE of eluted protein on a 10% acrylamide gel. For strains LAF-003, LAF-007 and LAF-009 hydrogenase protein is indicated through the presence of bands at 66 (Large) and 41 (Small) kDa. For strains LAF-010 and LAF-011 there is no clear band at 66 kDa and only faint bands at 41 kDa.

3. Assay method

All assays were performed in an anaerobic glovebox (Faircrest) filled with N_2. A H_2 gas flow of 100 scc min^{-1} (controlled by a Sierra SmartTrack 50 mass flow controller) was bubbled through a solution of 0.1 mM methylene blue in pH 4.5 “mixed hydrogenase” buffer (see below) for a minimum of 1 hour. Enzyme solution was diluted 100-fold in the same buffer. 2 mL of the methylene blue solution was then placed inside a 3 mL cuvette containing a 1 mm magnetic bead, and the cuvette was placed in an LED spectrophotometer (built in-house, Department of Chemistry Electronic Workshops, University of York) on top of magnetic stirrer plate, which was turned on. A light blocking cuvette lid with a small injection hole was placed over the cuvette, and the solution was illuminated at a wavelength of 626 nm for 150 sec, before injecting 50 µL of diluted enzyme solution. The absorbance was then measured until the methylene blue had been completely decolourised. An in-house determined methylene blue molar extinction coefficient of 28,000 µM^{-1} cm^{-1} was used to convert the fastest rate of absorbance change into H_2 oxidation rates, with protein concentrations determined as described above. The quoted assay rates are calculated averaging three repeat experiments.

The composition of the “mixed hydrogenase” buffer system is TAPS (N-tris(Hydroxymethyl)methyl-3-aminopropanesulfonic acid sodium-potassium salt) (Sigma Aldrich), HEPES (4-(2-hydroxyethyl)-1-piperazineethanesulfonic acid) (Sigma Aldrich), MES (2-(N-morpholino)ethanesulfonic acid) (Sigma Aldrich), and CHES (N-Cyclohexyl-2-aminoethanesulfonic acid) (AMRESCO) each at a 15 mM concentration, NaCl at 100 mM and deionised water. The pH of the buffer was adjusted using concentrated HCl and NaOH.

4. Electrochemistry method

All protein film electrochemistry was performed in an anaerobic glovebox (Faircrest), filled with nitrogen using previously described methods.[11] A three-electrode configuration was used in a gas-tight glass cell. The graphite working electrode and platinum wire counter electrode were housed in the water-jacketed main body of the cell. The same “mixed hydrogenase” buffer solution described in the assay section (above) was used in all the electrochemical measurements at sufficient volume to cover the electrode connections. A reference side arm at ambient temperature, filled with 100 mM NaCl, connected to the main cell by a Luggin capillary, housed the saturated calomel reference electrode. Gases (BOC) were flowed through the experimental setup at the stated composition, with constant total rate of 100 scc min^{-1} (where scc is standard cubic cm) under the control of mass flow controllers (Smart-Trak; Sierra Installations) connected to the electrochemical cell.

The graphite electrode surface (electrodes manufactured in-house) was prepared for enzyme application by sanding with Norton P1200 abrasive sheets before application of 2 µl of enzyme which was adsorbed onto the surface for 30 s. Excess film was removed by abrasion with cotton wool before insertion of the electrode into the electrochemical cell. The working electrode was rotated using an Origatrod rotator (Origalys) at 4000 rev min^{-1} to allow an adequate supply of substrate and removal of product. A CompactStat potentiostat (Ivium Technologies) and the IviumSoft program were used to control the experiment.

Other experimental conditions are as reported for each data set.

A reference electrode correction factor of E(V vs SHE) = E(V vs Ref) + 0.265 V was determined from 1 mM methylene blue cyclic voltammetry calibration measurements at pH 7, 25 °C, using a platinum working electrode and a value of $E_{m,7}$ = +19 mV vs SHE, calculated from published[12] reference data.

5. EPR spectroscopy Method

Small-volume EPR samples were prepared under anaerobic conditions in a Braun UniLab-plus glovebox (O_2 < 0.5 ppm, N_2 atmosphere), as described previously.[13] 250 µL of the Hyd-1 E73Q (approx. 12 µM, in 50 mM HEPES, 50 mM sodium phosphate, 150 mM sodium chloride, 30% (v/v) glycerol) was added to a water-jacketed glass electrochemical cell equipped with a 2 mm glass-encased Pt working electrode (both from Scientific Glassblowing Service, University of Southampton) and a Ag/AgCl mini-reference electrode (DRIREF-2, WPI). The enzyme solution, maintained at approximately 4°C, was stirred continuously and the potential was monitored throughout the titration (EmSTAT3+, PalmSens). Redox mediators (1,2 naphthoquinone, phenazine methosulfate, methylene blue, indigotrisulfonate, 2-hydroxy-1,4-naphthoquinone, benzyl viologen, methyl viologen) were added at a concentration of 30 µM each. The solution was adjusted to the desired potential by successive additions (0.1 – 0.5 µL) of sodium dithionite or potassium ferricyanide using a microlitre syringe (SGE Analytical Science) and 9 µL aliquots of the resultant enzyme-containing solution were successively transferred to a 1.6 O.D. quartz EPR tube (Wilmad). EPR samples were flash-frozen in the glove box cold finger containing EtOH, cooled externally using a dry-ice/acetone bath. Frozen samples were transferred to liquid nitrogen for storage.

EPR measurements were performed using an X/Q-band Bruker Elexsys E580 Spectrometer (Bruker BioSpin GmbH, Germany) equipped with a closed-cycle cryostat (Cryogenic Ltd, UK). The magnetic field was calibrated at room temperature with a Bruker strong pitch sample (g = 2.0028). All measurements were carried out at 20 K in an X-band split-ring resonator module with 2 mm sample access (ER 4118X-MS-2W). The Q value, as reported by the built-in Q indicator in the *Xepr* programme (typically 700) as well as the microwave frequency was used as a guide to position each sample in the same position in the resonator. EPR measurement conditions were 2 mW power, 100 kHz modulation frequency and 1.0 mT modulation amplitude for all samples. The Nernst plot (Fig. 4) was obtained by plotting the height of the EPR signal at g = 1.97 (maximal intensity normalised to 1) as a function of potential. The reduction potential (+211 ± 10 mV) varies slightly depending on which peak of the superoxidised proximal cluster EPR signal is monitored as a function of potential. The individual EPR spectra are shown in Fig. S9.

6. *Escherichia coli* hydrogenase-1 E73A variant electrochemistry

Experiments on the E73A variant confirm that making this amino acid exchange in *Escherichia coli* hydrogenase-1 has the same effect as was previously reported[11] in a study on *Salmonella* hydrogenase-5. As summarised in Fig. S3, the catalytic activity of E73A is comparable to Native enzyme (top panels), while the O_2 tolerance is seriously impaired (lower panels). Note that the experimental conditions for the O_2 inhibition measurements were chosen to match those in the *Salmonella* study.

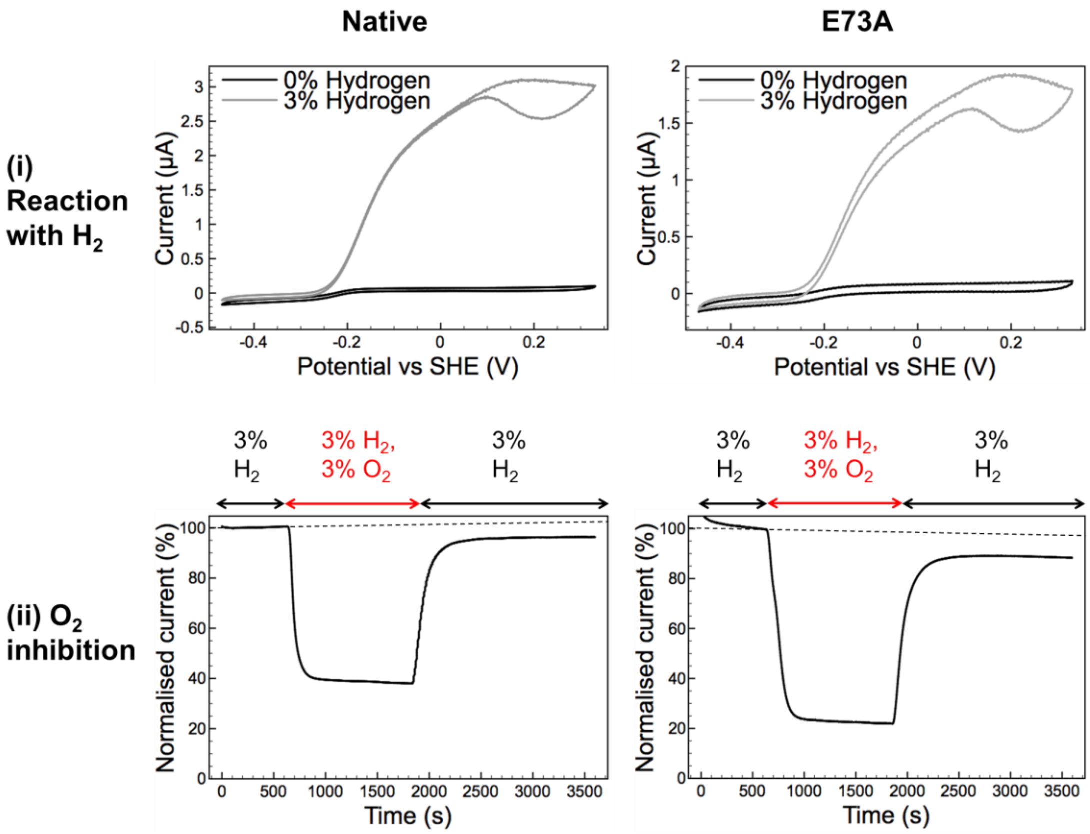

Fig. S3 (Top) Cyclic voltammograms measured under 3% H_2 (grey lines) and 0% H_2 (black lines) at 5 mVs^{-1}, pH 4.5, 37 °C for Native *Escherichia coli* Hyd-1 (left) and the E73A variant (right). (Bottom) Chronoamperometry experiments showing the extent of O_2 inhibition for Native (left) and the E73A variant (right) at pH 6.0 and -0.06 V vs SHE. The gas compositions are indicated above the graph and the current is normalised to the H_2 oxidation current measured just prior to O_2 addition. Other experimental conditions: electrode rotation rate 3000 rpm; carrier gas N_2 and total gas flow rate 100 scc min^{-1}.

7. The impact of $p(H_2)$ on enzyme activity

In order to determine the Michaelis constant for H_2 and the product inhibition constant, enzyme was adsorbed onto the electrode and then the cyclic voltammograms shown in Fig. S4 were measured at 5 mV s^{-1} under different concentrations of H_2.

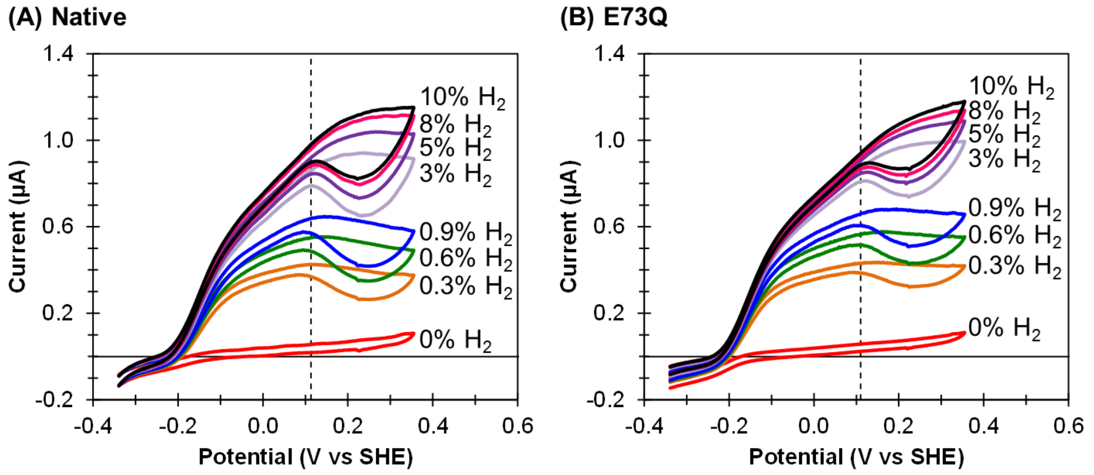

Fig. S4 Cyclic voltammograms measured under different H_2 and N_2 gas mixtures, as indicated, at 5 mVs^{-1}, pH 4.5, 37 °C for (A) Native *Escherichia coli* Hyd-1 and (B) the E73Q variant. The vertical dashed lines indicate +0.113 V vs SHE, the E_{mid} for methylene blue at pH 4.5. Other experimental conditions: electrode rotation rate 3500 rpm; carrier gas N_2 and total gas flow rate 100 scc min^{-1}.

Fig. S5A shows the Hanes-Woolf plot used to determine the Michaelis constant for H_2 for both Native and E73Q variant *Escherichia coli* hydrogenase-1, following the previously published procedure.[9] Fig. S5B shows analysis of the product inhibition constant for H_2 production, again using previously published methodology.[9] The data in Fig. S5 has been extracted from the voltammograms in Fig. S4. Tables S3 and S4 summarise the results of analysing repeat voltammogram experiments.

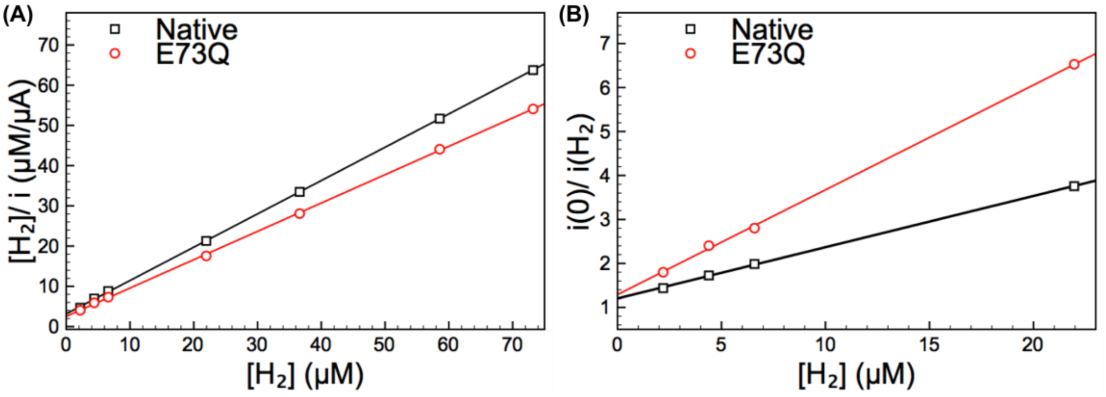

Fig. S5 (A) Hanes-Woolf analysis to determine the Michaelis constant for H_2 of both Native *Escherichia coli* Hyd-1 (black squares) and E73Q (red triangles) at +0.113 V vs SHE. (B) Inhibition plot to determine the inhibition constant for H_2 at -0.285 V vs SHE. Data extracted from the voltammograms shown in Fig. S4. The experiments were repeated four times each.

Table S3 Experimental repeats for the Hanes-Woolf Plot

Experiment	Gradient	Intercept	Calculated K_M
Native 1	1.02	3.99	3.9
Native 2	0.43	2.91	6.8
Native 3	0.85	2.62	3.1
Native 4	1.02	3.12	3.1
E73Q 1	0.72	1.73	2.4
E73Q 2	0.52	2.41	4.6
E73Q 3	1.05	3.30	3.1
E73Q 4	0.53	2.65	5.0

Table S4 Experimental repeats for the K_i plot

Experiment	Gradient	Intercept	Calculated K_i
Native 1	0.14	1.04	7.2
Native 2	0.16	1.26	6.3
Native 3	0.10	1.25	10.4
Native 4	0.07	1.27	13.9
E73Q 1	0.37	1.44	2.7
E73Q 2	0.16	1.42	6.4
E73Q 3	0.20	1.16	5.0
E73Q 4	0.23	1.14	4.3

8. The impact of pH on enzyme activity

Two different types of experiment were conducted to compare the impact of pH on Native *Escherichia coli* hydrogenase-1 and the E73Q variant. Fig. S6 shows 5 mVs^{-1} voltammograms measured by adsorbing enzyme onto the electrode and measuring activity at pH 4.5 under both 3% and 0% H_2, the pH was then changed to 6.0 and the same measurements were repeated, before a final buffer exchange to pH 7.6, and experiments in both 3% and 0% H_2. To overcome complications due to enzyme desorption, the voltammograms are normalised relative to the maximum current recorded under 3% H_2 at each pH.

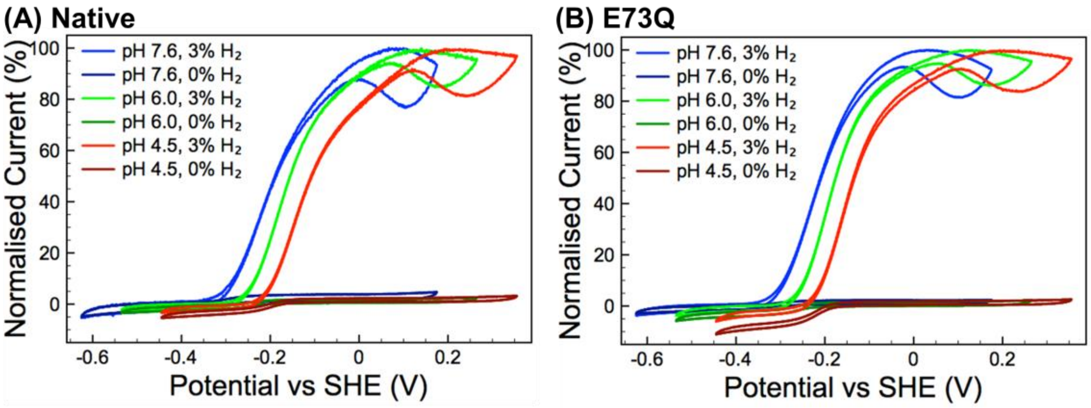

Fig. S6 Impact of pH of the enzymatic activity of (A) Native *Escherichia coli* Hyd-1 and (B) E73Q in both 0% and 3% H_2. Currents are normalised to the maximum value measured under 3% H_2 in each pH. Other experimental conditions: 5 mVs^{-1}, 37 °C, electrode rotation rate 3000 rpm; carrier gas N_2 and total gas flow rate 100 scc min^{-1}.

Accurate measurements of Eswitch, the potential at which the maximum rate of reductive reactivation of the Ni-B state occurs,[14] are not possible from the experiments shown in Fig. S6 and therefore the measurements shown in Fig. S7 were made. Fig. S8 summarises data extracted from both Fig. S6 and Fig. S7; the “Eonset” provides a measure of the impact of the E73Q amino acid exchange on the catalytic overpotential of the enzyme,[15] while the “Eswitch” data shows that the Native and E73Q enzymes do not differ in their reactivation profile.

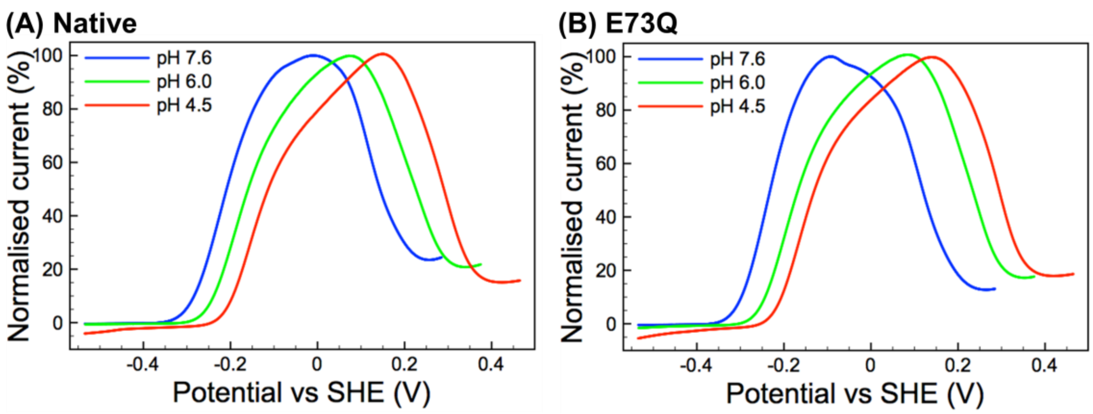

Fig. S7 The potential was held at +0.465 V vs SHE in pH 4.5, or +0.375 V vs SHE in pH 6.0, or +0.285 V vs SHE in pH 7.6 for 5 hours before the potential was scanned to -0.535 V vs SHE at a scan rate of 0.1 mVs^{-1}. Other conditions: 3% H_2 in N_2 with total gas flow rate 100 scc min^{-1}; electrode rotation rate 3000 rpm; 37 °C.

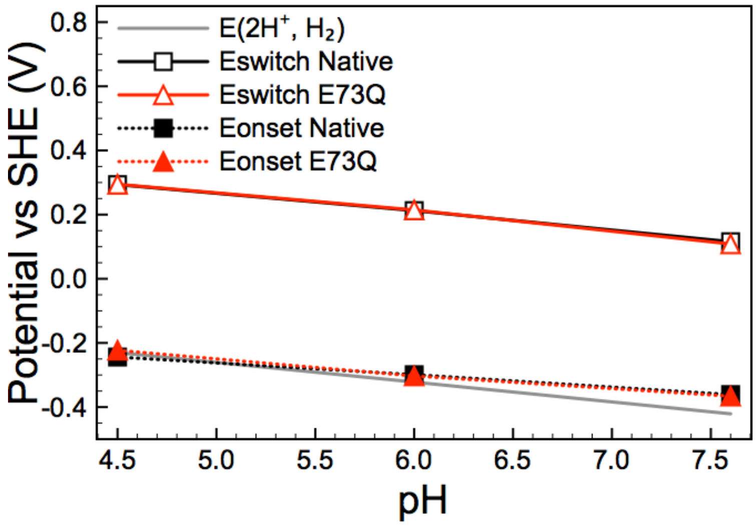

Fig. S8 Filled shapes show how the potential at which the current crosses the zero-axis (Eonset) in the 3% H_2 cyclic voltammograms in Fig. S5 changes relative to the reduction potential for the $2H^+/H_2$ couple calculated from the Nernst equation (E($2H^+$, H_2), grey line). Empty shapes show how Eswitch (determined from the maxima in the first derivative plot of experimental data shown in Fig. S6) changes as a function of pH.

9. EPR spectra

The EPR spectra which were analysed in order to extract the proximal cluster high-potential redox-transition midpoint potential are shown in Fig. S9. These experiments were conducted as detailed in the relevant methods section above.

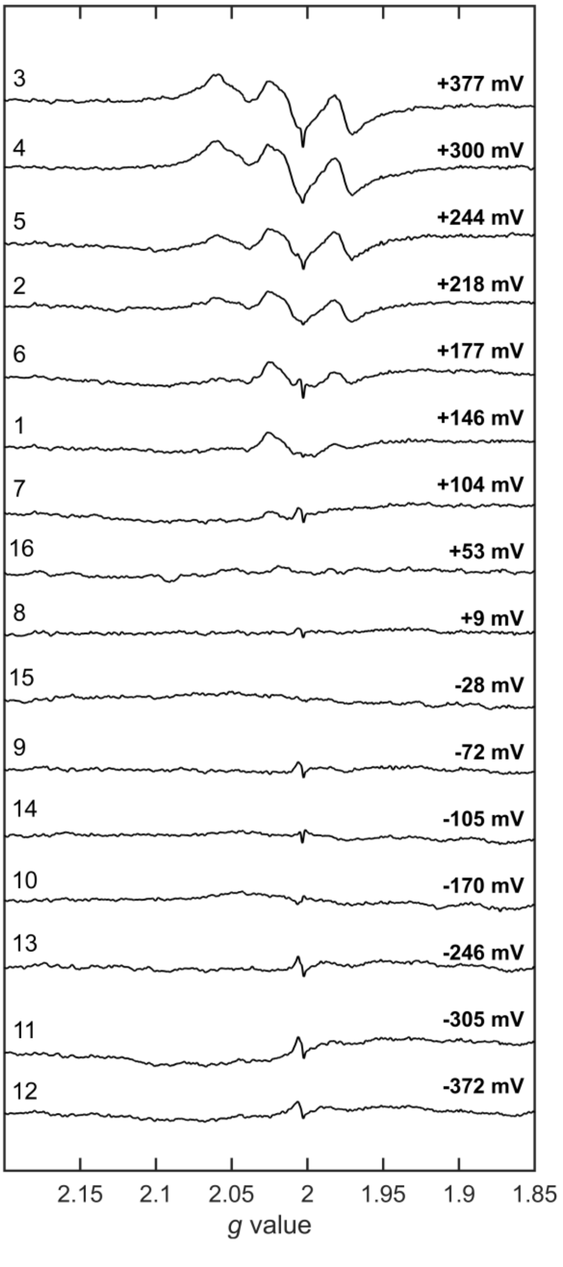

Fig. S9 Potentiometric titration of Hyd-1 E73Q. Small-volume (9 µL) EPR samples were prepared at fixed potentials under anaerobic conditions, as described in section 5. The order in which samples were taken is also indicated by the numbers on the left. All EPR measurements were carried out at 20 K with 2 mW power, 100 kHz modulation frequency and 1.0 mT modulation amplitude. All spectra were baseline subtracted.

References

1. R. M. Evans, A. Parkin, M. M. Roessler, B. J. Murphy, H. Adamson, M. J. Lukey, F. Sargent, A. Volbeda, J. C. Fontecilla-Camps and F. A. Armstrong, *J. Am. Chem. Soc.*, 2013, **135**, 2694.
2. A. Parkin, in *The Metal-Driven Biogeochemistry of Gaseous Compounds in the Environment*, eds. P. M. H. Kroneck and M. E. S. Torres, Springer Netherlands, 2014, vol. 14, p. 99.
3. W. Lubitz, H. Ogata, O. Rüdiger and E. Reijerse, *Chem. Rev.*, 2014, **114**, 4081.
4. A. Parkin and F. Sargent, *Current Opinion in Chemical Biology*, 2012, **16**, 26.
5. R. Heerman, T. Zeppenfeld and K. Jung, *Microbial Cell Factories*, 2008, **7**, 7.
6. D. Hanahan, *Journal of Molecular Biology*, 1983, **166**, 557-580.
7. K. Hayashi, N. Morooka, Y. Yamamoto, K. Fujita, K. Isono, S. Choi, E. Ohtsubo, T. Baba, B. L. Wanner, H. Mori and T. Horiuchi, *Mol. Syst. Biol.*, 2006, **2**.
8. M. J. Casadaban and S. N. Cohen, *J. Mol. Biol.*, 1980, **138**, 179.
9. M. J. Lukey, A. Parkin, M. M. Roessler, B. J. Murphy, J. Harmer, T. Palmer, F. Sargent and F. A. Armstrong, *J. Biol. Chem.*, 2010, **285**, 3928.
10. E. Gasteiger, C. Hoogland, A. Gattiker, S. Duvaud, M. R. Wilkins, R. D. Appel and A. Bairoch, in *The Proteomics Protocols Handbook*, ed. J. M. Walker, Humana Press, 2005, pp. 571-607.
11. L. Bowman, L. Flanagan, P. K. Fyfe, A. Parkin, W. N. Hunter and F. Sargent, *Biochem. J.*, 2014, **458**, 449.
12. A. Hulanicki and S. Glab, *Pure & Appl. Chem.*, 1978, **5-**, 463.
13. J. J. Wright, E. Salvadori, H. Bridges, J. Hirst and M. M. Roessler, Invited Paper, Under review in Journal of Inorganic Biochemistry.
14. V. Fourmond, P. Infossi, M.-T. Giudici-Orticoni, P. Bertrand and C. Léger, *J. Am. Chem. Soc.*, 2010, **132**, 4848.
15. B. J. Murphy, F. Sargent and F. A. Armstrong, *Energy Environ. Sci.*, 2014, **7**, 1426–1433.
